# Management of immune checkpoint inhibitor‐related adverse events: A review of case reports

**DOI:** 10.1111/1759-7714.13315

**Published:** 2020-01-22

**Authors:** Xiaoyan Si, Peng Song, Jun Ni, Mingyi Di, Chunxia He, Li Zhang, Xiaowei Liu, Yue Li, Hanping Wang, Xiaoxiao Guo, Jiaxin Zhou, Lian Duan, Xu Yang, Mengzhao Wang, Li Zhang

**Affiliations:** ^1^ Department of Pulmonary and Critical Care Medicine Peking Union Medical College Hospital Beijing China; ^2^ Department of Dermatology Peking Union Medical College Hospital Beijing China; ^3^ Department of Clinical Laboratory Peking Union Medical College Hospital Beijing China; ^4^ Department of Ophthalmology Peking Union Medical College Hospital Beijing China; ^5^ Department of Gastroenterology Peking Union Medical College Hospital Beijing China; ^6^ Department of Cardiology Peking Union Medical College Hospital Beijing China; ^7^ Department of Rheumatology Peking Union Medical College Hospital Beijing China; ^8^ Department of Endocrinology Peking Union Medical College Hospital Beijing China; ^9^ Department of Liver Surgery Peking Union Medical College Hospital Beijing China

**Keywords:** Adverse event, immune checkpoint inhibitor, immunotherapy, toxicity

## Abstract

Immune checkpoint inhibitors represent a major breakthrough in cancer therapy. Immune‐related adverse events (irAEs) may occur during treatment due to their unique mechanism of action. Management of irAEs is based on clinical experience because it is not easy to conduct prospective trials to evaluate the best treatment strategy. Using a combination of search terms in the PubMed and Embase databases, we reviewed all cases in the English language citing toxicities associated with either pembrolizumab, nivolumab, ipilimumab, atezolizumab, tremelimumab, durvalumab, avelumab or any combination of these agents published before 20 May 2019. A total of 128 reports with 239 cases were included in the study. Here, we summarize the spectrum of toxicities, safety in special patients, rechallenging after irAEs and agents used for treatment of irAEs in those reports.

## Key points

This work summarizes the spectrum of irAEs, safety in special patients, rechallenging after irAEs and agents used for treatment of irAEs in 128 case reports.

## Introduction

Immune checkpoint inhibitors (ICIs) represent a major breakthrough in cancer therapy. Immune‐related adverse events (irAEs) may occur during treatment due to their unique mechanism of action. IrAEs are generally manageable but can be fatal in some cases.^1^ Management of irAEs is based on clinical experience because it is not easy to conduct prospective trials, although professional groups have developed guidelines of management. Using a combination of research terms in the PubMed and Embase databases, we reviewed all cases in the English language citing toxicities associated with either pembrolizumab, nivolumab, ipilimumab, atezolizumab, tremelimumab, durvalumab, avelumab or any combination of these agents published before 20 May 2019. A total of 128 reports with 239 cases were included in the study. Here, we summarize the spectrum of toxicities, safety in special patients, rechallenging after irAEs and agents used for treatment of irAEs in those cases.

## Toxicity profile

IrAEs occur in up to 90% of patients treated with an anti‐CTLA‐4 antibody and 70% of patients treated with a PD‐1/PD‐L1 antibody.^2^ The profile of irAE was different for PD‐1/PD‐L1 inhibitors and CTLA‐4 inhibitors. Several organ specific AE rates varied among tumor sites. The most frequent AEs of any grade with PD‐1/PD‐L1 inhibitors and CTLA‐4 inhibitors alone were diarrhea (11% and 36%), fatigue (21% and 25%) pruritus (15% and 25%) and rash (10% and 23%).^3^ The frequency of colitis ranged from 8% to 22%. It was reported that hypophysitis can affect up to 10% of patients treated with anti‐CTLA‐4 inhibitors.^2^ Hepatitis occurred in 5% to 10% of patients during treatment with ipilimumab, nivolumab and pembrolizumab.^4^ Thyroid dysfunction occurred in 5% to 10% patients receiving PD‐1/PD‐L1 inhibitors. Pneumonitis occurred in around 1% of patients treated with PD‐1/PD‐L1 or CTLA‐4 inhibitors.^2^

IrAEs can mimic autoimmune diseases and affect any organ system. irAEs in the case reports included in the study are summarized in Table 1. Besides common toxicities in different systems, the case reports also describe rare toxicities.

**Table 1:** Spectrum of immune‐related adverse events in case reports

System	Immune‐related adverse events (number of case reports)
Dermatologic (32)	Vitiligo (1), granuloma annulare (2), bullous pemphigoid (3), psoriasis (22), erythema multiforme (1), lichenoid reaction (2), Grover's disease (1)
Endocrinologic (52)	Type 1 diabetes mellitus (38), hypophysitis (9), isolated adrenal insufficiency (1), thyroid storm (2), hypothyroidism (2)
Gastrointestinal (47)	Acute liver failure (1), hepatitis (8), bile duct obstruction (1), cholangitis (1), pancreatitis (1), hemorrhagic gastritis (1), ileitis (1), colitis (32), intestinal obstruction (1)
Pulmonary (11)	Organizing pneumonia (5), sarcoidosis (2), pneumonitis (4)
Neurologic (20)	Myasthenia gravis (5), Guillain‐Barre syndrome (3), cerebral edema (1), necrotizing encephalopathy (1), encephalitis (2), mononeuropathy multiplex with rhabdomyolysis (1), necrotic myelopathy (1), Bell's palsy (1), inflammation enteric neuropathy (1), brachial plexus neuritis (2), peripheral neuropathy (2)
Cardiac (7)	Myocarditis (2), cardiomyopathy (1), coronary spasm (1), pericardial effusion (3)
Rheumatologic (28)	Remitting seronegative symmetrical synovitis with pitting edema (1), arthritis (12), dermatomyositis/myositis (4), Goodpasture's disease (1), scleroderma (2), polymyalgia rheumatic (3), sicca syndrome (5)
Nephrotic (7)	Cystitis (1), renal failure (1), nephrotic syndrome (4), acute glomerulonephritis (1)
Hematologic (16)	Pancytopenia (2), neutropenia (6), aplastic anemia (2), pure red cell aplasia (1), thrombocytopenia (3), acute thrombosis (1), hemophagocytic lymphohistiocytosis (1)
Ophthalmologic (17)	Uveitis (7), Vogt‐Koyanagi‐Harada disease‐like uveitis (2), orbital inflammation (3), dry eye (2), ulcerative keratitis (1), ocular myositis (2)
Otorhinolaryngologic (2)	Sinusitis (2)

The most frequent skin irAEs reported were rash and pruritus. Vitiligo, depigmented macules resulting from the loss of melanocytes, occurred mostly in melanoma patients treated with ICIs, while it also occurred in lung cancer patients.^5^ The PD‐L1/PD‐1 pathway probably mediates peripheral tolerance of melanosomal proteins, and PD‐1 inhibitor may induce vitiligo.^6^ Grover's disease, which presents as an intensely pruritic, papulovesicular rash, is a rare dermatologic toxicity. It has occasionally been reported during treatment with ipilimumab.^7^ It has been suggested that Th2 cells may play a possible role in its pathogenesis, and systemic corticosteroids may improve pruritus.

It was reported that hypophysitis occurred mostly in patients treated with anticytotoxic T lymphocyte associated antigen‐4 (CTLA4) inhibitors due to CTLA‐4 expressed on pituitary cells.^8^ However, hypophysitis has also been reported in patients treated with atezolizumab or nivolumab.^9^ Hypophysitis induced by PD‐1/PD‐L1 inhibitor usually presents as isolated ACTH deficiency. The pituitary gland can be divided into two different parts: the anterior and posterior lobes. The anterior lobe of the pituitary gland is made up of several different types of cells that produce and release different types of hormones, including growth hormone, thyroid‐stimulating hormone (TSH), adrenocorticotropic hormone (ACTH), follicle‐stimulating hormone, luteinizing hormone and prolactin. The order of hormone deficiency specific to hypophysitis is as follows: ACTH > TSH > luteinizing hormone/follicle‐stimulating hormone > prolactin > growth hormone.^10^ Immune‐related hypophysitis may not affect all pituitary cell lines, and isolated ACTH deficiency has been reported.^9^, ^11^

ICI‐induced bile duct obstruction and cholangitis have been reported, and they should be considered as causes of cholangitis.^12^ Patients presented with fever, abdominal discomfort, fatigue, predominant elevation of alkaline phosphatase (ALP) and gamma‐glutamyl‐transpeptidase (GGT), and moderate elevation of transaminases (ALT/AST) and total bilirubin. Liver biopsy showed a predominant CD8‐positive T cell infiltrate. The response to corticosteroids was disappointing.^13^

Sarcoidosis presenting with hilar and mediastinal lymphadenopathy was reported in a lung cancer patient who received carboplatin/pemetrexed and pembrolizumab.^14^ T helper‐1 and T helper‐17 cells may play a role in the development of sarcoidosis.^15^ It is important to take a biopsy to differentiate sarcoidosis and disease progression. Most patients required discontinuation of therapy completely for a short period of time as well as immunosuppressant treatment.

While cardiac irAEs are rare, ICI‐induced myocarditis has a high fatality rate.^1^ Coronary spasm was reported in a patient treated with nivolumab.^16^ It was found that exhausted T cells expressing programmed cell death protein 1 (PD‐1) existed in atherosclerotic plaques. Therefore, treatment with PD‐1 inhibitors may have unanticipated consequences in cancer patients with underlying cardiovascular disease.^17^

Most patients with inflammatory arthritis from ICIs were negative for rheumatoid factor and anticyclic citrullinated peptide antibodies traditionally associated with rheumatoid arthritis.^18^ Treatment of inflammatory arthritis included nonsteroidal anti‐inflammatory drugs (NSAIDs), prednisone, and both nonbiologic and biologic disease‐modifying antirheumatic drugs (DMARDs).^19^

Hematological irAEs, including autoimmune hemolytic anemia, immune thrombocytopenia, neutropenia and aplastic anemia, are rarely reported. Surveillance of the complete blood count is essential. Oncologists should keep ICIs in mind as a possible cause of monolineage cytopenia or pancytopenia. Acute thrombosis was reported in a lung cancer patient treated with pembrolizumab.^20^ As coagulation disorders are common in cancer patients, it was not certain that the thrombosis could be attributed to pembrolizumab.

Patients presenting with photosensitivity, blurred vision, lacrimation, and diplopia, need an ophthalmology referral, although ophthalmic irAEs, such as uveitis, orbital inflammatory, and ulcerative keratitis, are rare.^21^, ^22^ Most patients were treated with topical or systemic corticosteroids.

Two cases of sinusitis, presenting as sinus pressure and nasal congestion after treatment of nivolumab, were reported to have responded to anti‐TNF therapy.^23^

## Safety of ICIs in patients with immune system impairment

Patients with pre‐existing diseases, such as autoimmune diseases and human immunodeficiency virus (HIV) infection were excluded from most clinical trials of ICIs. Safety of ICIs in this population is unclear.

It was suggested that patients with autoimmune disease could benefit from immunotherapy and experience tolerable toxicities that are manageable with immunosuppressive regimens. But close clinical monitoring is essential.^24^ Calabrese *et al*. reported a patient with psoriatic arthritis who experienced a psoriasis flare 2.8 weeks after starting nivolumab, while the patient with rheumatoid arthritis remained without disease activity on hydroxychloroquine throughout his course of immunotherapy.^25^ Uemura *et al*. reported a patient with advanced melanoma and refractory Crohn's disease who was treated concurrently with pembrolizumab and tocilizumab, which did not result in Crohn's disease exacerbation. It suggests that targeted immunosuppression combined with checkpoint inhibitors may be a treatment strategy for patients with autoimmune diseases.^26^

It was reported that highly active antiretroviral therapy improved the survival of patients with HIV, which increased the incidence of malignancies.^27^ Li *et al*. reported that a patient with HIV infection and non‐small cell lung cancer was treated with pembrolizumab and SBRT, and then experienced massive pericardial effusion and interstitial pneumonia.^28^ However, this case failed to explain the relationship between HIV infection and AEs. ICIs for the treatment of advanced‐stage cancer in patients with HIV infection might be associated with no new safety signals.^29^

## Rechallenging with ICIs after irAEs

Whether patients who experienced irAEs should be rechallenged with immunotherapy is a question for clinicians. When a good response (complete or partial) is observed prior to the onset of irAEs that require a treatment delay, it may be better to suspend retreatment with ICIs. A total of 15 patients were rechallenged with ICIs after experiencing irAEs details of which are provided in Table 2. Recurrence of irAEs is possible during rechallenging. Guidelines recommend permanent discontinuation of ICIs following a CTCAE grade 4 toxicity except endocrine toxicities which can be treated with hormone replacement.

**Table 2:** Rechallenging after immune‐related adverse events (irAEs) in case reports

Reports	Sex/age	Malignancy	Immunotherapy	irAEs	Management of irAEs	Retreatment	Outcomes
Utsunomiya *et al*.^30^	F/37	Melanoma	Nivolumab, ipilimumab	Erythema multiforme major	Systemic corticosteroid, IVIG‡	Nivolumab	Not described
Anastasopoulou *et al*.^32^	M/48	Melanoma	Nivolumab	Bullous pemphigoid	Systemic corticosteroids	Pembrolizumab, ipilimumab	No recurrence
Uemura *et al*.^7^	M/73	Melanoma	Ipilimumab	Grover's disease	Systemic corticosteroids	Ipilimumab	Recurrence
Kanie *et al*.^9^	M/65	NSCLC†	Atezolizumab	Hypophysitis	Hydrocortisone replacement	Atezolizumab	No recurrence
Chae *et al*.^33^	M/76	NSCLC†	Pembrolizumab	Diabetes mellitus	Insulin	Pembrolizumab	No recurrence
Imafuku *et al*.^31^	M/62	Melanoma	Nivolumab	Pneumonitis	Systemic corticosteroids	Nivolumab	No recurrence
	M/75	Melanoma	Nivolumab	Pneumonitis	Systemic corticosteroids	Nivolumab	Recurrence
Shaheen *et al*.^34^	F/70	NSCLC†	Nivolumab	Pericardial effusion	Systemic corticosteroids	Nivolumab	No recurrence
Abu Samra *et al*.^35^	M/82	Melanoma	Pembrolizumab	Uveitis	Steroid eye drops	Pembrolizumab	Recurrence
Theillac *et al*.^22^	M/55	Melanoma	Nivolumab	Uveitis	Systemic corticosteroids, steroid eye drops	Nivolumab	No recurrence
Papavasileiou *et al*.^36^	F/54	Melanoma	Ipilimumab	Uveitis	Topical steroid	Ipilimumab	Hypophysitis, colitis
	F/47	Melanoma	Ipilimumab	Orbital inflammation	Systemic corticosteroids	Ipilimumab	Recurrence
Nguyen *et al*.^37^	M/55	Melanoma	Nivolumab	Dry eye, corneal perforation	Topical cyclosporine, topical loteprednol, autologous serum tears, doxycycline	Nivolumab	No recurrence
Ngo *et al*.^38^	M/70	Melanoma	Nivolumab, ipilimumab	Synovitis	Systemic corticosteroids	Nivolumab	No recurrence
du Rusquec *et al*.^39^	F/77	Melanoma	Ipilimumab	Pancytopenia	Systemic corticosteroids, IVIG‡, erythropoietin, filgrastim	Ipilimumab	Recurrence

† Non‐small cell lung cancer.

‡ Intravenous immunoglobulin.

Utsunomiya *et al*. reported a patient retreated with nivolumab after grade 4 erythema multiforme major.^30^ However, they did not recommend retreatment after grade 4 dermatologic toxicities according to NCCN and ESMO guidelines. For cases of interstitial pneumonitis consistent with a diffuse alveolar damage (DAD) pattern, it is suggested that the corticosteroid dose should be gradually reduced over time, and ICIs should be discontinued.^31^

A retrospective study showed the risk‐reward ratio for an anti‐PD‐1 or anti‐PD‐L1 rechallenge appeared to be acceptable, although these patients require close monitoring.^40^

## Agents used for treatment of irAEs

Most irAEs are steroid‐sensitive and resolve within six to 12 weeks. If irAEs show insufficient improvement despite the use of adequate corticosteroids, immunomodulatory agents should be considered after exclusion of other causes.

Intravenous immunoglobulin (IVIg) has been used with corticosteroid in patients with immunotherapy‐related erythema multiforme major,^30^ myasthenia gravis,^41^ Guillain‐Barré syndrome,^42^ encephalopathy,^43^ peripheral neuropathy,^44^ scleroderma,^45^ ocular myositis,^46^ pancytopenia,^39^, ^47^ and neutropenia.^48^ Most irAEs showed improvement, while myasthenia gravis, Guillain‐Barré syndrome, and necrotizing encephalopathy worsened in some patients.

Infliximab is a chimeric monoclonal antibody binding to tumor necrosis factor‐α. Infliximab was used in patients with immunotherapy‐related polymyalgia rheumatic,^25^ peripheral neuropathy,^44^ necrotic myelopathy,^43^ Guillain‐Barré syndrome,^49^ hemorrhagic gastritis,^50^ and organizing pneumonia.^51^ Infliximab is recommended to be used in patients with severe immunotherapy‐related toxicities whose symptoms cannot be controlled by corticosteroids within 48 to 72 hours according to the National Comprehensive Cancer Network (NCCN) guidelines.^52^ However, Abu‐Sbeih *et al*. reported that infliximab should be introduced early in the disease course of immunotherapy‐related colitis instead of waiting until failure of corticosteroid therapy or corticosteroid taper.^53^

Adalimumab is a recombinant human monoclonal antibody that binds specifically to tumor necrosis factor‐α, blocking interaction with its cell surface receptors and thereby reducing the impact of inflammation. Adalimumab was used in two cases of sinusitis induced by ICIs.^23^

Tocilizumab is a recombinant humanized anti‐human interleukin 6 (IL‐6) receptor monoclonal antibody. Tocilizumab was used in patients with immunotherapy‐related pneumonitis^54^ and arthritis.^55^

Rituximab is a chimeric monoclonal antibody binding to CD20 proteins. Rituximab was used in patients with autoimmune encephalitis associated with nivolumab and ipilimumab.^56^

Martins *et al*. proposed a so‐called shut‐off strategy aimed at inhibiting key inflammatory components involved in the pathophysiological processes of irAEs, and limited potential adverse effects of drug immunosuppression on tumor response.^57^ The biological immunosuppressive agents are important to manage refractory irAEs.

## Limitations of review

The limitations of this review are potential selection bias and publication bias based on case reports. Authors and editors usually choose rare and successfully managed cases to publish. Our study is limited by information available in the original reports. This review did not include cases of immunotherapies combined with chemotherapy, which may cause a higher incidence and severity of irAEs.

In conclusion, as immune‐related toxicity can affect any organ system, clinicians should keep this in mind as a possible cause of any symptom or abnormality during treatment of ICIs. Rechallenging with ICIs after irAEs requires close monitoring. The biological immunosuppressive agents will be important to manage refractory irAEs. Further research establishing optimal guidelines on how to manage irAEs is necessary.

## Disclosure

The authors have no potential conflicts of interest to disclose.

## References

[1] Wang DY , Salem JE , Cohen JV *et al* Fatal toxic effects associated with immune checkpoint inhibitors: A systematic review and meta‐analysis. JAMA Oncol 2018; 4 (12): 1721–8. 10.1001/jamaoncol.2018.3923. 30242316 PMC6440712

[2] Michot JM , Bigenwald C , Champiat S *et al* Immune‐related adverse events with immune checkpoint blockade: A comprehensive review. Eur J Cancer 2016; 54: 139–48. 10.1016/j.ejca.2015.11.016. 26765102

[3] Arnaud‐Coffin P , Maillet D , Gan HK *et al* A systematic review of adverse events in randomized trials assessing immune checkpoint inhibitors. Int J Cancer 2019; 145 (3): 639–48. 10.1002/ijc.32132. 30653255

[4] Haanen J , Carbonnel F , Robert C *et al* Management of toxicities from immunotherapy: ESMO Clinical Practice Guidelines for diagnosis, treatment and follow‐up. Ann Oncol 2017; 28 (suppl_4): iv119–iv42. 10.1093/annonc/mdx225. 28881921

[5] Uenami T , Hosono Y , Ishijima M *et al* Vitiligo in a patient with lung adenocarcinoma treated with nivolumab: A case report. Lung Cancer 2017; 109: 42–4. 10.1016/j.lungcan.2017.04.019. 28577948

[6] Tewalt EF , Cohen JN , Rouhani SJ *et al* Lymphatic endothelial cells induce tolerance via PD‐L1 and lack of costimulation leading to high‐level PD‐1 expression on CD8 T cells. Blood 2012; 120 (24): 4772–82. 10.1182/blood-2012-04-427013. 22993390 PMC3520619

[7] Uemura M , Fa'ak F , Haymaker C *et al* A case report of Grover's disease from immunotherapy‐a skin toxicity induced by inhibition of CTLA‐4 but not PD‐1. J Immunother Cancer 2016; 4: 55 10.1186/s40425-016-0157-6. 27660709 PMC5028978

[8] Postow MA , Sidlow R , Hellmann MD . Immune‐related adverse events associated with immune checkpoint blockade. N Engl J Med 2018; 378 (2): 158–68. 10.1056/NEJMra1703481. 29320654

[9] Kanie K , Iguchi G , Bando H *et al* Two cases of atezolizumab‐induced hypophysitis. J Endocr Soc 2018; 2 (1): 91–5. 10.1210/js.2017-00414. 29362727 PMC5774896

[10] Fukuoka H . Hypophysitis. Endocrinol Metab Clin North Am 2015; 44 (1): 143–9. 10.1016/j.ecl.2014.10.011. 25732650

[11] Kotwal A , Rao S , Haas RA . Ipilimumab‐induced hypophysitis may not affect all pituitary cell lines: A case report. J Endocrinol Metab 2015; 5 (5): 299–303. 10.14740/jem287w.

[12] Kashima J , Okuma Y , Shimizuguchi R , Chiba K . Bile duct obstruction in a patient treated with nivolumab as second‐line chemotherapy for advanced non‐small‐cell lung cancer: A case report. Cancer Immunol Immunother 2018; 67 (1): 61–5. 10.1007/s00262-017-2062-3. 28913619 PMC11028312

[13] Gelsomino F , Vitale G , Ardizzoni A . A case of nivolumab‐related cholangitis and literature review: How to look for the right tools for a correct diagnosis of this rare immune‐related adverse event. Invest New Drugs 2018; 36 (1): 144–6. 10.1007/s10637-017-0484-6. 28631096

[14] Fakhri G , Akel R , Salem Z , Tawil A , Tfayli A . Pulmonary sarcoidosis activation following neoadjuvant pembrolizumab plus chemotherapy combination therapy in a patient with non‐small cell lung cancer: A case report. Case Rep Oncol 2017; 10 (3): 1070–5. 10.1159/000484596. 29515398 PMC5836160

[15] Rambhia PH , Reichert B , Scott JF *et al* Immune checkpoint inhibitor‐induced sarcoidosis‐like granulomas. Int J Clin Oncol 2019; 24 (10): 1171–81. 10.1007/s10147-019-01490-2. 31321613

[16] Ferreira M , Pichon E , Carmier D *et al* Coronary toxicities of anti‐PD‐1 and anti‐PD‐L1 immunotherapies: A case report and review of the literature and international registries. Target Oncol 2018; 13 (4): 509–15. 10.1007/s11523-018-0579-9. 30006825

[17] Fernandez DM , Rahman AH , Fernandez NF *et al* Single‐cell immune landscape of human atherosclerotic plaques. Nat Med 2019; 25 (10): 1576–88. 10.1038/s41591-019-0590-4. 31591603 PMC7318784

[18] Haikal A , Borba E , Khaja T , Doolittle G , Schmidt P . Nivolumab‐induced new‐onset seronegative rheumatoid arthritis in a patient with advanced metastatic melanoma: A case report and literature review. Avicenna J Med 2018; 8 (1): 34–6. 10.4103/ajm.AJM_127_17. 29404271 PMC5782418

[19] Weinmann SC , Pisetsky DS . Mechanisms of immune‐related adverse events during the treatment of cancer with immune checkpoint inhibitors. Rheumatology (Oxford) 2019; 58 (Suppl. 7): vii59–67. 10.1093/rheumatology/kez308. 31816080 PMC6900913

[20] Kunimasa K , Nishino K , Kimura M *et al* Pembrolizumab‐induced acute thrombosis: A case report. Medicine (Baltimore) 2018; 97 (20): e10772 10.1097/MD.0000000000010772. 29768369 PMC5976311

[21] Hanna KS . A rare case of pembrolizumab‐induced uveitis in a patient with metastatic melanoma. Pharmacotherapy 2016; 36 (11): e183–e88. 10.1002/phar.1839. 27716999

[22] Theillac C , Straub M , Breton AL , Thomas L , Dalle S . Bilateral uveitis and macular edema induced by Nivolumab: A case report. BMC Ophthalmol 2017; 17 (1): 227 10.1186/s12886-017-0611-3. 29195497 PMC5709862

[23] Dein E , Sharfman W , Kim J *et al* Two cases of sinusitis induced by immune checkpoint inhibition. J Immunother 2017; 40 (8): 312–4. 10.1097/CJI.0000000000000174. 28614096 PMC5593775

[24] Johnson DB , Beckermann KE , Wang DY . Immune checkpoint inhibitor therapy in patients with autoimmune disease. Oncology 2018; 32 (4): 190–4. 29684232

[25] Calabrese C , Kirchner E , Kontzias A , Velcheti V , Calabrese LH Rheumatic immune‐related adverse events of checkpoint therapy for cancer: Case series of a new nosological entity. RMD Open 2017; 3 (1): e000412 10.1136/rmdopen-2016-000412. 28405474 PMC5372131

[26] Uemura M , Trinh VA , Haymaker C *et al* Selective inhibition of autoimmune exacerbation while preserving the anti‐tumor clinical benefit using IL‐6 blockade in a patient with advanced melanoma and Crohn's disease: A case report. J Hematol Oncol 2016; 9 (1): 81 10.1186/s13045-016-0309-7. 27595932 PMC5011857

[27] Sigel K , Makinson A , Thaler J . Lung cancer in persons with HIV. Curr Opin HIV AIDS 2017; 12 (1): 31–8. 10.1097/coh.0000000000000326. 27607596 PMC5241551

[28] Li D , He C , Xia Y , Du Y , Zhang J . Pembrolizumab combined with stereotactic body radiotherapy in a patient with human immunodeficiency virus and advanced non‐small cell lung cancer: A case report. J Med Case Reports 2018; 12 (1): 104 10.1186/s13256-018-1667-2. PMC5911965 29681240

[29] Cook MR , Kim C . Safety and efficacy of immune checkpoint inhibitor therapy in patients with HIV infection and advanced‐stage cancer: A systematic review. JAMA Oncol 2019; 5 (7): 1049–54. 10.1001/jamaoncol.2018.6737. 30730549

[30] Utsunomiya A , Oyama N , Iino S *et al* A case of erythema multiforme major developed after sequential use of two immune checkpoint inhibitors, nivolumab and ipilimumab, for advanced melanoma: Possible implication of synergistic and/or complementary immunomodulatory effects. Case Rep Dermatol 2018; 10 (1): 1–6. 10.1159/000485910. 29515387 PMC5836162

[31] Imafuku K , Yoshino K , Yamaguchi K , Tsuboi S , Ohara K , Hata H . Two cases of nivolumab re‐administration after pneumonitis as immune‐related adverse events. Case Rep Oncol 2017; 10 (1): 296–300. 10.1159/000463379. 28512413 PMC5422733

[32] Anastasopoulou A , Papaxoinis G , Diamantopoulos P *et al* Bullous pemphigoid‐like skin lesions and overt eosinophilia in a patient with melanoma treated with nivolumab: Case report and review of the literature. J Immunother 2018; 41 (3): 164–7. 10.1097/cji.0000000000000210. 29309291

[33] Chae YK , Chiec L , Mohindra N , Gentzler R , Patel J , Giles F . A case of pembrolizumab‐induced type‐1 diabetes mellitus and discussion of immune checkpoint inhibitor‐induced type 1 diabetes. Cancer Immunol Immunother 2017; 66 (1): 25–32. 10.1007/s00262-016-1913-7. 27761609 PMC11028603

[34] Shaheen S , Mirshahidi H , Nagaraj G , Hsueh CT . Conservative management of nivolumab‐induced pericardial effusion: A case report and review of literature. Exp Hematol Oncol 2018; 7: 11 10.1186/s40164-018-0104-y. 29761026 PMC5941729

[35] Abu Samra K , Valdes‐Navarro M , Lee S , Swan R , Foster CS , Anesi SD . A case of bilateral uveitis and papillitis in a patient treated with pembrolizumab. Eur J Ophthalmol 2016; 26 (3): e46–8. 10.5301/ejo.5000724. 26692064

[36] Papavasileiou E , Prasad S , Freitag SK , Sobrin L , Lobo AM . Ipilimumab‐induced ocular and orbital inflammation–a case series and review of the literature. Ocul Immunol Inflamm 2016; 24 (2): 140–6. 10.3109/09273948.2014.1001858. 25760920

[37] Nguyen AT , Elia M , Materin MA , Sznol M , Chow J . Cyclosporine for dry eye associated with nivolumab: A case progressing to corneal perforation. Cornea 2016; 35 (3): 399–401. 10.1097/ico.0000000000000724. 26771550

[38] Ngo L , Miller E , Valen P , Gertner E . Nivolumab induced remitting seronegative symmetrical synovitis with pitting edema in a patient with melanoma: A case report. J Med Case Reports 2018; 12 (1): 48 10.1186/s13256-018-1579-1. PMC6389137 29478412

[39] du Rusquec P , Saint‐Jean M , Brocard A *et al* Ipilimumab‐induced autoimmune pancytopenia in a case of metastatic melanoma. J Immunother 2014; 37 (6): 348–50. 10.1097/cji.0000000000000041. 24911795

[40] Simonaggio A , Michot JM , Voisin AL *et al* Evaluation of readministration of immune checkpoint inhibitors after immune‐related adverse events in patients with cancer. JAMA Oncol 2019; 5: 1310 10.1001/jamaoncol.2019.1022. PMC6555478 31169866

[41] March KL , Samarin MJ , Sodhi A , Owens RE . Pembrolizumab‐induced myasthenia gravis: A fatal case report. J Oncol Pharm Pract 2018; 24 (2): 146–9. 10.1177/1078155216687389. 28147928

[42] Tanaka R , Maruyama H , Tomidokoro Y *et al* Nivolumab‐induced chronic inflammatory demyelinating polyradiculoneuropathy mimicking rapid‐onset Guillain‐Barré syndrome: A case report. Jpn J Clin Oncol 2016; 46 (9): 875–8. 10.1093/jjco/hyw090. 27380808

[43] Abdallah AO , Herlopian A , Ravilla R *et al* Ipilimumab‐induced necrotic myelopathy in a patient with metastatic melanoma: A case report and review of literature. J Oncol Pharm Pract 2016; 22 (3): 537–42. 10.1177/1078155215572932. 25712627

[44] Thaipisuttikul I , Chapman P , Avila EK . Peripheral neuropathy associated with ipilimumab: A report of 2 cases. J Immunother 2015; 38 (2): 77–9. 10.1097/CJI.0000000000000070. 25658617 PMC5628623

[45] Barbosa NS , Wetter DA , Wieland CN , Shenoy NK , Markovic SN , Thanarajasingam U . Scleroderma induced by pembrolizumab: A case series. Mayo Clin Proc 2017; 92 (7): 1158–63. 10.1016/j.mayocp.2017.03.016. 28599746

[46] Pushkarevskaya A , Neuberger U , Dimitrakopoulou‐Strauss A , Enk A , Hassel JC . Severe ocular myositis after ipilimumab treatment for melanoma: A report of 2 cases. J Immunother 2017; 40 (7): 282–5. 10.1097/cji.0000000000000178. 28604554

[47] Atwal D , Joshi KP , Ravilla R , Mahmoud F . Pembrolizumab‐induced pancytopenia: A case report. Perm J 2017; 21: 17–004. 10.7812/TPP/17-004. PMC5528855 28746020

[48] Barbacki A , Maliha PG , Hudson M , Small D . A case of severe pembrolizumab‐induced neutropenia. Anticancer Drugs 2018; 29 (8): 817–9. 10.1097/CAD.0000000000000661. 29889673

[49] Rutzen‐Lopez IM , Fu JB , Arias‐Berrios JE . Poster 362: Nivolumab‐induced concurrent Guillain‐Barré syndrome and myasthenia gravis in a patient with metastatic renal cell carcinoma: A case report. P M and R ‐ The Journal of Injury, Function and Rehabilitation 2017; 9: S246–S47. 10.1016/j.pmrj.2017.08.301.

[50] Cinnor B , Crossman H , Kaplan J , Mittal C , Gerich ME , Kao DJ . First reported case of pembrolizumab‐induced immune mediated hemorrhagic gastritis. Gastroenterology 2017; 152 (5): S891 10.1016/s0016-5085(17)33042-1.

[51] Ortega Sanchez G , Jahn K , Savic S , Zippelius A , Läubli H . Treatment of mycophenolate‐resistant immune‐related organizing pneumonia with infliximab. J Immunother Cancer 2018; 6 (1): 85 10.1186/s40425-018-0400-4. 30176946 PMC6122461

[52] Thompson JA , Schneider BJ , Brahmer J *et al* Management of immunotherapy‐related toxicities, version 1.2019. J Natl Compr Canc Netw 2019; 17 (3): 255–89. 10.6004/jnccn.2019.0013. 30865922

[53] Abu‐Sbeih H , Ali FS , Wang X *et al* Early introduction of selective immunosuppressive therapy associated with favorable clinical outcomes in patients with immune checkpoint inhibitor‐induced colitis. J Immunother Cancer 2019; 7 (1): 93 10.1186/s40425-019-0577-1. 30940209 PMC6444537

[54] Naqash AR , Yang LV , Sanderlin EJ , Atwell DC , Walker PR . Interleukin‐6 as one of the potential mediators of immune‐related adverse events in non‐small cell lung cancer patients treated with immune checkpoint blockade: Evidence from a case report. Acta Oncol 2018; 57 (5): 705–8. 10.1080/0284186x.2017.1406668. 29171332

[55] Kim ST , Tayar J , Trinh VA *et al* Successful treatment of arthritis induced by checkpoint inhibitors with tocilizumab: A case series. Ann Rheum Dis 2017; 76 (12): 2061–4. 10.1136/annrheumdis-2017-211560. 28830882

[56] Williams TJ , Benavides DR , Patrice KA *et al* Association of autoimmune encephalitis with combined immune checkpoint inhibitor treatment for metastatic cancer. JAMA Neurol 2016; 73 (8): 928–33. 10.1001/jamaneurol.2016.1399. 27271951

[57] Martins F , Sykiotis GP , Maillard M *et al* New therapeutic perspectives to manage refractory immune checkpoint‐related toxicities. Lancet Oncol 2019; 20 (1): e54–64. 10.1016/S1470-2045(18)30828-3. 30614479

